# Effects of Trimethylamine Concentrations in Hatching Eggs on Chick Quality in Dwarf Hens

**DOI:** 10.3390/ani15142121

**Published:** 2025-07-17

**Authors:** Xuefeng Shi, Lin Xuan, Jiahui Lai, Caiyun Jiang, Junying Li, Guiyun Xu, Jiangxia Zheng

**Affiliations:** 1 College of Animal Science and Technology, China Agricultural University, Beijing 100193, China; xuefengshi@alu.cau.edu.cn (X.S.); linxuan@cau.edu.cn (L.X.); iamljhui@163.com (J.L.); cyjiang@cau.edu.cn (C.J.); lijunying@cau.edu.cn (J.L.); ncppt@cau.edu.cn (G.X.); 2 College of Animal Science and Technology, Guangxi University, Nanning 530004, China

**Keywords:** TMA, chick quality, Pasgar score, chick hatchability, growth curve

## Abstract

Microbial contamination of hatching eggs often leads to reduced hatchability and poor chick quality. As trimethylamine (TMA), a metabolite derived from dietary choline, has antimicrobial properties, increasing yolk TMA contents may increase bacterial resistance to eggs; however, the effects of TMA concentrations on chick quality remain unknown. Hence, this study was conducted to determine the effects of yolk TMA concentrations on the hatchability and chick quality of dwarf hens with different *FMO3* genotypes. Hens (n = 140) were divided into control and experimental groups; the latter received choline chloride (2800 mg/kg) to elevate their yolk TMA concentrations. The TMA content, Pasgar score, hatchability, and post-hatching performance were evaluated. The results showed that choline supplementation significantly increased TMA concentrations in hens with AT and TT genotypes. Higher yolk TMA concentrations (≥4 µg/g) correlated with improved Pasgar scores and reduced abnormalities in vitality, navel, and yolk sac absorption. Hatchability peaked at 6.49 µg/g TMA, suggesting a threshold effect. Although the growth rate remained unaffected, chick mortality decreased in the high-TMA group. Therefore, moderate TMA concentrations can enhance egg antimicrobial defenses and improve reproductive performance. This strategy provides a biologically grounded alternative to traditional chemical disinfection in hatcheries.

## Simple Summary

Ensuring the successful hatching of healthy chicks and improving chick quality is crucial for the poultry industry. Chemical disinfectants have been traditionally used to protect hatching eggs from bacterial infection; however, these methods can harm the developing embryos and pollute the environment. In this study, we explored a safer and more natural approach of increasing the concentration of trimethylamine (TMA), which has antibacterial properties in eggs. We fed dwarf hens a specific dietary supplement to increase TMA concentrations in egg yolk and studied the effects on hatching eggs. The results show that eggs with a higher TMA had improved hatching rates and produced stronger, healthier chicks with fewer physical defects. Furthermore, these chicks exhibited lower mortality rates in early life. Our method offers a promising new approach by enhancing the antibacterial components inside hatching eggs, thereby improving chick survival and quality while potentially reducing reliance on external chemical treatments.

## 1. Introduction

Bacterial contamination of hatching eggs is a critical issue in poultry production, as it contributes to decreased hatchability, increased early embryonic mortality, and poor chick quality, resulting in substantial economic losses for commercial hatcheries [1,2,3,4]. Pathogenic bacteria such as *Salmonella* spp., *Mycoplasma* spp., and *Escherichia coli* can penetrate the eggshell and infect the egg contents—especially resulting in yolk sac infection—during storage or incubation, posing a serious threat to embryonic development [5,6,7].

Commercial hatcheries commonly employ various external disinfection methods to reduce microbial contamination of hatching eggs, such as formaldehyde fumigation, hydrogen peroxide spraying, ultraviolet irradiation, and ozone treatment [4,8,9]. While these approaches are effective in reducing surface bacterial loads, they also have drawbacks. Formaldehyde, for instance, is a known irritant and potential carcinogen, posing health and environmental concerns [10]. Hydrogen peroxide and ozone require precise control of exposure and equipment, and ultraviolet treatment may be ineffective in eggs with surface irregularities. Furthermore, all of these methods act on the shell surface and do not eliminate microorganisms that may have already penetrated the shell. In this study, we explore an internal antimicrobial strategy based on the accumulation of trimethylamine (TMA) in the egg yolk, induced by dietary choline supplementation in hens carrying a mutation in the flavin-containing monooxygenase 3 (*FMO3)* gene. This approach may enhance the eggs’ intrinsic antimicrobial capacity and reduce the need for external chemical disinfection.

TMA accumulation was induced by dietary choline supplementation in hens carrying a T329S mutation (FMO3 c.984 A > T) in the flavin-containing monooxygenase 3 (*FMO3*) gene [11,12]. This mutation reduces FMO3 enzyme activity, impairing its ability to oxidize TMA into trimethylamine-N-oxide (TMAO), and thereby leads to an increased TMA content in eggs [13,14].

The deposition of TMA and well-known antimicrobials such as lysozymes in the egg yolk is part of the egg’s antimicrobial system [15]. Furthermore, TMA concentrations reflect the amount of antibacterial substances that have been replaced in eggs with increasing storage time. For instance, Li et al. [16] discovered that although the humid egg-laying environment of ducks poses a substantial threat to incubation and promotes the proliferation of pathogenic microorganisms, the high TMA content of duck eggs (1.5 times higher than that of chicken eggs) makes them resistant to bacterial infections and improves their reproductive capacity [16]. However, research exploring the potential effects of the elevated TMA content in hatching eggs on their incubation performance and chick quality has not been conducted to date.

Therefore, the objective of this study was to determine whether dietary choline supplementation in hens with the FMO3 T329S mutation, resulting in increased TMA deposition in egg yolks, could enhance hatching performance and improve chick quality. We hypothesized that TMA-enriched eggs exhibit stronger intrinsic antimicrobial properties, leading to improved embryo survival and chick development.

## 2. Materials and Methods

### 2.1. Experimental Materials and Feeding Management

The experiment was conducted at the Experimental Unit for Poultry Genetic Resources and Breeding of China Agricultural University, China. Prior to the experiment, blood samples were collected from 65-week-old dwarf hens for *FMO3* genotyping. The genotypes of individual *FMO3* genes (AA, AT, and TT) were determined by performing polymerase chain reaction-restriction fragment length polymorphism [11]. A total of 45 AA-type, 270 AT-type, and 165 TT-type hens were identified; of these, 140 healthy *FMO3*-genotyped hens (40 AA, 50 AT, and 50 TT) were selected for the experiment. Based on their genotypes, the hens were randomly and equally divided into control (C_AA, C_AT, C_TT) and experimental groups (E_AA, E_AT, E_TT). The control group was fed a basal diet (Table 1), whereas the experimental group received the same basal diet supplemented with 2800 mg of choline chloride per kilogram. The dosage was chosen based on Wang et al. [11], where 2800 mg/kg choline chloride effectively elevated yolk TMA concentrations without negatively affecting hen performance.

All hens were housed individually in conventional laying hen cages in the same environmentally controlled poultry house. Each cage was equipped with a trough feeder and nipple drinker. Hens were subjected to a 16 h/8 h light/dark cycle per day. Feed and water were provided ad libitum. Each hen was housed in an individual standard laying cage with dimensions of 45 cm (length) × 40 cm (width) × 45 cm (height), providing a floor area of 0.18 m^2^ per hen.

### 2.2. Collection of Hatching Eggs

The hens were reared for 5 weeks and artificially inseminated twice weekly starting from the third week. Artificial insemination was performed using 100 µL of freshly collected semen per hen. Semen was obtained from healthy dwarf roosters of the same genetic line, pooled from multiple individuals, and used immediately without dilution. Hatching eggs were collected, while abnormal eggs (such as cracked, de-formed, and damaged) were discarded in the fourth week. After this screening, a total of 582 fertilized eggs were selected for incubation during the experimental period (C_AA: 70 eggs, C_AT: 106 eggs, C_TT: 110 eggs, E_AA: 80 eggs, E_AT: 101 eggs, and E_TT: 115 eggs). These eggs were collected over a period of 5 days. During storage prior to incubation, the eggs were kept at a temperature of 16–18 °C and relative humidity of 65–75%.

### 2.3. Determination of the TMA Content of the Egg Yolk

Yolk TMA concentrations were determined using headspace gas chromatography with slight modifications based on the method by Wang et al. [11]. In each group, yolks from ten eggs were used for TMA analysis. Briefly, 15 g of mixed yolk was homogenized with 20 mL of 10% (*w*/*v*) trichloroacetic acid, kept at room temperature overnight, and filtered. A 2 mL filtrate was transferred into a 10 mL vial, followed by addition of 0.5 mL of 10% formaldehyde, 5 mL of toluene, and 1.5 mL of 50% KOH. The vial was sealed with a Teflon-lined septum and incubated at 60 °C for 30 min. Headspace gas (500 μL) was analyzed using a 7890A gas chromatograph (Agilent Technologies, Palo Alto, CA, USA) equipped with a flame ionization detector and a DB-Wax capillary column (30 m × 0.32 mm, 0.25-μm film). The oven was programmed from 40 °C (3 min) to 180 °C at 10 °C/min, held for 4 min; the injector was kept at 200 °C and the detector at 250 °C, the split ratio was 10:1, and hydrogen at 1 mL/min was used as the carrier gas.

### 2.4. Hatching

After removing defective eggs, hatching eggs were disinfected using formaldehyde fumigation (30 mL 40% formaldehyde + 15 g KMnO_4_ per m^3^) at 25–28 °C for 20 min. Eggs were incubated in a single model incubator (EIFDMS-16800; Qingdao, China) at 37.8 °C and 60% humidity, automatically turned every 2 h until day 18, and then transferred to hatching baskets. Chicks hatched on day 21. Each chick was individually leg-banded with unique numbers for identification.

### 2.5. Hatchability and Chick Scoring

Chick quality was assessed using the Pasgar scoring system, which evaluates five criteria. The scoring standards are summarized in Table 2 [17].

### 2.6. Assessment of Chick Growth Performance

Chick body weight was recorded at hatch. Following the chick quality assessment, the chicks were reared under standard brooding conditions. The initial stocking density was approximately 35 chicks/m^2^ and was gradually reduced to 20 chicks/m^2^ by week 3. Continuous lighting (24 h) was provided during the first 48 h post-hatch, followed by a 18 h/6 h light/dark cycle. The ambient temperature was maintained at 33–35 °C during the first week and gradually reduced by 2–3 °C per week until reaching 24 °C. All chicks had ad libitum access to feed and water. A commercial corn–soybean meal-based starter diet was provided, formulated to meet or exceed National Research Council (1994) requirements, containing approximately 20.5% crude protein and 2850 kcal/kg metabolizable energy. Body weight and chick mortality were recorded at 1, 2, 3, 5, and 8 weeks of age.

### 2.7. Classification Based on Yolk TMA Concentration

To further investigate the influence of yolk TMA concentrations on chick hatchability and quality, hatching eggs were categorized into two groups based on their average yolk TMA content: high-TMA (≥4 μg/g) and low-TMA groups (≤4 μg/g). The high-TMA group mainly included the E_AT and E_TT groups, while the low-TMA group included the C_AA, C_AT, C_TT, and E_AA groups.

### 2.8. Correlation Analysis Between TMA Content and Chick Development Indicators

To further evaluate the effect of yolk TMA contents on chick health, the average TMA content, hatchability, hatch weight, and mortality rate during the brooding period of the chicks in the control and experimental groups were subjected to correlation analyses.

Furthermore, the relationship between yolk TMA concentrations and hatchability was modeled using quadratic polynomial regression:

hatchability (%) = 60.5 + 8.52 × TMA − 0.656 × TMA^2,^ (1)

where TMA represents the TMA concentration in egg yolk (μg/g). The regression model demonstrated strong explanatory power (*R*^2^ = 0.85).

### 2.9. Statistical Analysis

Descriptive statistics are presented as the mean ± standard error of the mean (SE). Linear or quadratic regression was performed to analyze and assess the relationships between the chick quality indicators. Differences among groups were analyzed using one-way analysis of variance, and *p*-values less than 0.05 were considered significant. Statistical analyses and data visualization were performed using SPSS (version 25.0, IBM Corp., Armonk, NY, USA) and R (version 4.3.2, R Foundation for Statistical Computing, Vienna, Austria), respectively.

## 3. Results

### 3.1. TMA Content of the Egg Yolk

**Figure 1:**
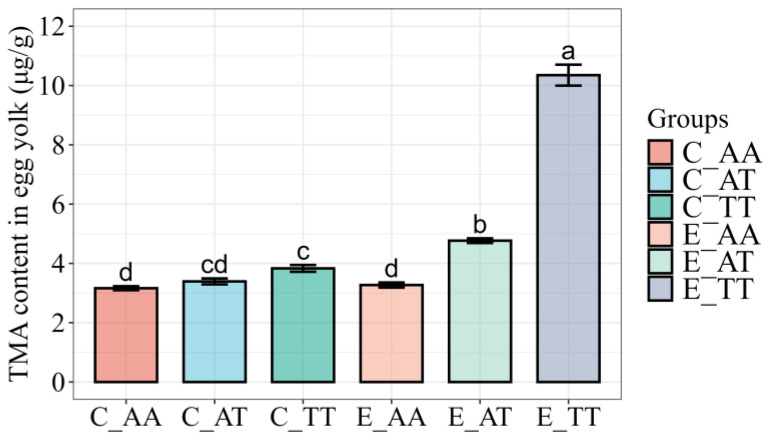
Variability in TMA concentrations in hatching eggs among the different groups. Different letters indicate significant differences (*p* < 0.05). Data are presented as the mean ± SE.

The TMA content of the egg yolks across the different groups is shown in Figure 1. The TMA content of the egg yolk was significantly lower in the C_AA group (3.16 ± 0.06 μg/g) than in the C_TT group (3.83 ± 0.11 μg/g; *p* < 0.05). Nonetheless, the TMA content of the egg yolk did not significantly differ between the C_AT (3.39 ± 0.10 μg/g) and C_TT groups or between the C_AT and C_AA groups. Moreover, all experimental subgroups (E_AA: 3.27 ± 0.11 μg/g; E_AT: 4.77 ± 0.08 μg/g; E_TT: 10.35 ± 0.34 μg/g) exhibited significant differences in the TMA content of the egg yolk (*p* < 0.05). Additionally, compared with their corresponding genotypes in the control group, the TMA content of the egg yolk significantly increased (*p* < 0.05) in the E_TT and E_AT groups, whereas that in the E_AA group did not exhibit any significant difference.

### 3.2. Pasgar Scores of Chicks

**Figure 2:**
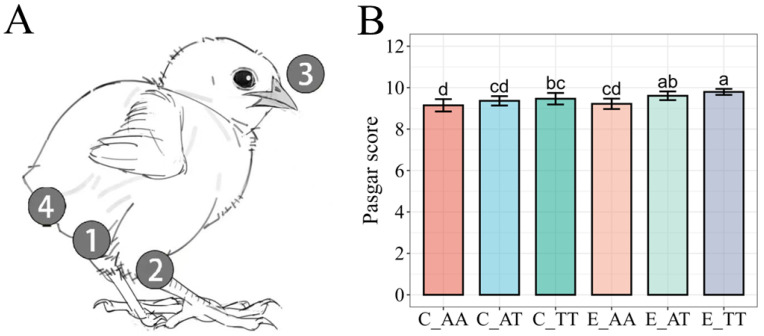
The Pasgar score evaluation in chicks. (**A**) Pasgar Scoring Schematic for Chicks. 1. Navel; 2. Legs; 3. Beak; 4. Belly. (**B**) Comparison of chick quality Pasgar scores among different groups. Bars labeled with different letters indicate significant differences (*p* < 0.05). The data are presented as mean ± SE.

Figure 2A shows a schematic of the Pasgar scoring for chicks, while Figure 2B presents the evaluation results for chick quality. Among the control subgroups, the Pasgar scores of the C_AA group (9.15 ± 0.15) were significantly lower than those of the C_TT group (9.46 ± 0.10, *p* < 0.05). However, no significant difference was observed between the C_AT (9.36 ± 0.09) and C_TT groups or between the C_AT and C_AA groups. Moreover, in the experimental subgroups, the Pasgar score of the E_AA group (9.21 ± 0.15) was significantly lower than those of the E_AT (9.61 ± 0.07) and E_TT groups (9.79 ± 0.01, *p* < 0.05); however, no significant differences were observed between the E_AT and E_TT groups. Additionally, the Pasgar scores of the E_TT and E_AT chicks were significantly higher (*p* < 0.05) than those of their control counterparts. However, Pasgar scores did not significantly differ between the E_AA and C_AA groups.

Table 3 shows the detailed results of egg hatchability and Pasgar scores of the chicks. The addition of choline to the daily diet of the laying hens increased the TMA content of the hatching eggs, resulting in reduced incidence of vitality issues, navel problems, and belly abnormalities in the newly hatched chicks. The Pasgar scores of the control group were lower than those of the experimental group, primarily due to suboptimal vitality (20.59% in C_AA, 3.64% in C_AT, and 2.74% in C_TT), navel (44.12% in C_AA, 21.82% in C_AT, and 17.81% in C_TT), and belly abnormality scores (2.94% in C_AA, 12.73% in C_AT, and 8.22% in C_TT).

### 3.3. Growth Curve and Mortality Rate of Chicks

**Figure 3:**
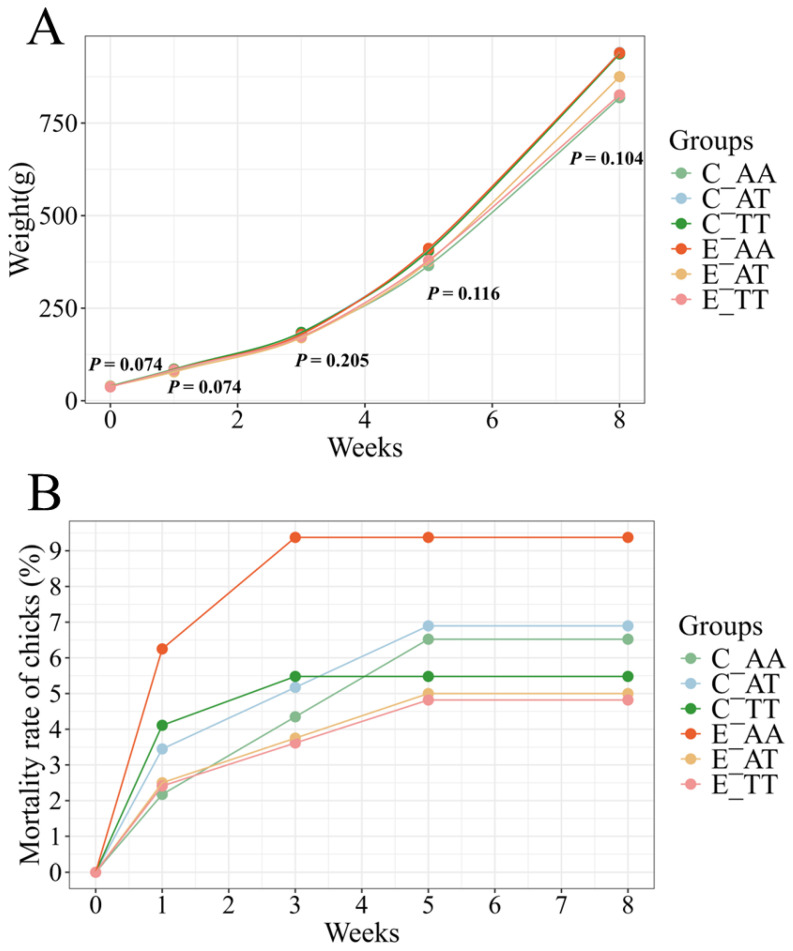
The growth performance of chicks. (**A**) Growth curves of chicks in the first 8 weeks. (**B**) The chick mortality rate in the first 8 weeks.

All chicks were fed a standard diet and monitored for body weight and mortality over the first 8 weeks after the Pasgar score evaluation. Body weight did not significantly differ among the groups (Figure 3A). In addition, the mortality rate of the chicks in all groups gradually increased over time and stabilized by the fifth week (Figure 3B). By the eighth week, the mortality rate of the E_AA chicks (9.37%) remained the highest, followed by that of the C_AT (6.70%), C_AA (6.52%), and C_TT (5.48%) chicks. Notably, the mortality rate of the E_AT (5.00%) and E_TT (4.82%) chicks was the lowest.

### 3.4. Comparison of Chick Quality Between High- and Low-TMA Groups

The high-TMA group had significantly higher Pasgar scores (9.63 ± 0.04 vs. 9.26 ± 0.07, *p* = 0.001) and a higher percentage of chicks with perfect Pasgar scores (75.30 ± 4.17% vs. 51.61 ± 5.35%, *p* = 0.028) than the low-TMA group (Table 4). Although the hatching rate of the high-TMA group (82.93 ± 2.27%) was slightly higher than that of the low-TMA group (81.22 ± 0.64%), this difference was not statistically significant (*p* = 0.535). Similarly, the rates of navel and belly abnormalities and abnormal vitality were lower in the high-TMA group than in the low-TMA group; however, these differences were not statistically significant. These results further support the notion that increased yolk TMA concentrations positively influence chick quality at hatching.

### 3.5. Results of Correlation Between TMA Content and Chick Development

**Figure 4:**
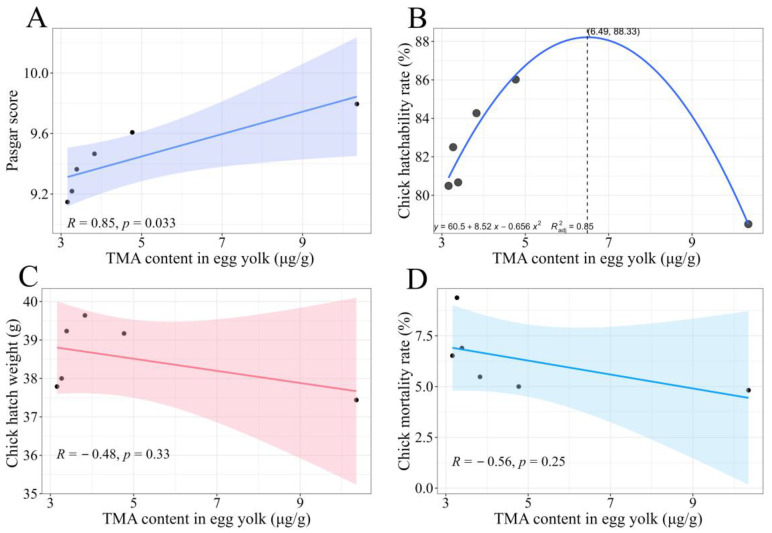
The correlation analysis results between the average TMA content in egg yolks, Pasgar score and hatchability chick quality. (**A**–**D**) depict the correlation between the average TMA content in egg yolks and Pasgar scores, chick hatchability rate, chick hatch weight, and chick mortality rate, respectively.

A strong positive correlation was observed between the yolk TMA content and Pasgar score (*R* = 0.85, *p* = 0.033), indicating that higher TMA concentrations are associated with better chick quality (Figure 4A).

The significance of the quadratic term (*p* = 0.028) confirmed a non-linear relationship between TMA concentrations and hatchability (Figure 4B). Hatchability increased with increasing TMA contents, reaching a peak of 88.33% at the optimal TMA concentration of 6.49 μg/g. However, further increases beyond this threshold led to a decline in hatchability. This finding suggests that although moderate elevation in yolk TMA contents enhances the egg’s antimicrobial defense, excessively high concentrations may impair embryonic development, possibly by disrupting yolk homeostasis or suppressing beneficial microbiota.

In contrast, no significant correlations were found between TMA contents and chick hatch weight (*R* = −0.48, *p* = 0.33) or mortality during brooding (*R* = −0.56, *p* = 0.25; Figure 4C,D), although an overall trend toward reduced mortality at higher TMA contents was observed.

## 4. Discussion

Our findings demonstrate that dietary choline supplementation can increase the TMA content of the yolk of hatching eggs. TMA metabolism is influenced by various factors, including diet, genetics, and gut microbiota composition [11,13,14,18]. At the genetic level, the FMO3 enzyme in the liver oxidizes TMA to TMAO [13]. When FMO3 enzyme activity decreases, TMA levels increase [13,19]. In terms of diet, poultry consume foods rich in lecithin, carnitine, betaine, creatinine, and choline, which promote TMA production in the body, leading to increased TMA accumulation in egg yolks [11,16]. TMA is an intestinal microorganism-dependent dietary choline metabolite [20,21]; the composition and abundance of the intestinal microflora affect TMA levels in the body [22,23]. In addition, some intestinal microorganisms possess TMA reductase activity and can reduce TMAO to TMA, thereby affecting the dynamic balance of TMA [24]. For instance, *Akkermansia* and *Mucispirillum*, the two major intestinal bacteria, exert negative and positive effects, respectively, on TMA metabolism [16]. In this study, we utilized dwarf hens carrying FMO3 mutations to investigate how dietary choline affects TMA concentrations in hatching eggs across different genotypes. As expected, eggs from the choline-supplemented E_AT and E_TT groups exhibited higher TMA concentrations, supporting our hypothesis and aligning with the results of previous studies [11]. These findings highlight a genotype-dependent response to choline that directly contributes to the yolk TMA content.

The elevated TMA content observed in our study may contribute to enhanced antimicrobial protection of hatching eggs, thereby improving egg quality during incubation. TMA is responsible for the fishy odor of poultry eggs [14]. However, Ward et al. [25] revealed that ordinary eggs contain only trace amounts of TMA (less than 4 μg/g in egg yolk), and most people will not perceive a fishy odor from them. In addition, TMA reduces plaque accumulation and exhibits antibacterial and anti-inflammatory properties [26,27]. TMA is part of the antibacterial system of poultry eggs and plays a significant role in protecting them from external microbial invasion [15,16]. Importantly, previous research has shown that TMA retains or even enhances its antimicrobial function as storage time increases, whereas the activity of other antimicrobial substances such as lysozyme or cuticle proteins declines [15]. This suggests that the increased TMA content in eggs from choline-supplemented hens may help maintain antimicrobial defense during prolonged incubation, which is particularly relevant for practical hatching conditions.

Our data further suggest that higher TMA concentrations in hatching eggs are associated with improved chick quality. Microbial contamination of hatching eggs can adversely affect hatchability and chick performance [10,28]. Jin et al. [29] investigated the microbial composition of egg yolks and whites during incubation. They found that egg whites were not sterile during incubation and that microorganisms from egg whites could migrate into egg yolks. Additionally, they identified *Muribaculaceae* and *Rothia* as a beneficial bacteria family and a harmful bacteria genus, respectively, in chicks [29]. Considering these findings, enhancing the antimicrobial properties of eggs by increasing TMA levels may reduce the risk of microbial infection during embryonic development, thereby supporting better chick health. In traditional egg production industries, poultry eggs are chemically disinfected prior to incubation [1,30]. However, this method not only causes environmental pollution but also poses issues such as absorption of disinfectants by the eggshell, which may come into contact with the embryo and result in embryonic mortality or decreased hatching rates [10,31]. Thus, dietary manipulation to elevate TMA contents could offer a safer and more sustainable strategy for improving chick quality without relying on external disinfection methods.

Although our findings suggest that elevated yolk TMA contents may contribute to internal antimicrobial defense and improved chick outcomes, we acknowledge that microbiological analyses during incubation were not performed—representing a limitation of our study. However, prior evidence supports this mechanism: Shi et al. [15] demonstrated that TMA inhibits *E. coli* and synergizes with lysozyme in eggs, while Li et al. [16] showed that eggs from FMO3-deficient ducks, which accumulate more TMA, exhibited stronger antibacterial activity. These results support the hypothesis that TMA forms part of an internal antimicrobial barrier within the egg. Future studies incorporating microbial profiling and challenge experiments are necessary to verify the role of TMA during embryogenesis.

While cleanliness practices maintain egg cleanliness, such measures cannot eliminate bacteria already present inside the eggs [3,32]. Our results suggest that chick vitality as well as navel and belly development are the primary factors influencing Pasgar scores. If the contents of the chick’s yolk sac are not fully absorbed, the weight of the internal organs will decrease, resulting in fewer maternal antibodies transferred from the yolk sac to the body, thus affecting chick growth and development [33]. Therefore, we propose that the reason for the reduced chick vitality is the failure to fully absorb nutrients in the yolk sac, resulting in stunted growth.

Microorganisms also affect egg hatchability. During incubation, external microorganisms enter the embryo through the eggshell, which affects the overall health of the embryo [1,29,34,35]. Interestingly, our analysis revealed a threshold effect between yolk TMA and hatchability: below 6.49 μg/g, TMA positively correlated with the hatch rate, whereas above this value, a negative correlation was observed. This suggests a dual role of TMA—moderate concentrations may suppress pathogenic microbes, while excessive concentrations may disrupt the balance of beneficial microbiota. This was further supported by our group comparison: although differences in overall hatchability were not statistically significant, the high-TMA group showed slightly better hatch rates and higher chick quality. These findings indicate that moderately increased TMA may promote embryonic development, while excessive concentrations could be detrimental.

Moreover, diseases are important factors contributing to the increased mortality rate of chicks during the brooding period [36,37]. We observed that the mortality rate of the E_AT and E_TT chicks was lower. This may be attributed to the absorption of higher concentrations of yolk-derived TMA, which may act in concert with maternal antibodies (e.g., IgY, IgM, and IgA) to provide early immune protection and enhance resistance to environmental challenges during the brooding period [38,39,40]. Notably, the laying hens used in this study were 65 weeks old. Although they were selected for their stable egg production and prior genotyping of the FMO3 mutation, hens at this age are generally associated with lower fertility and hatchability compared to younger flocks. This may have affected the overall incubation performance and limited the observable effects of dietary treatment on hatchability. Future studies should consider using younger hens to validate the findings under optimal reproductive conditions.

Furthermore, the TMA content of hatching eggs was not significantly correlated to chick mortality during brooding. However, this lack of significance may be attributed to sample size limitations. Nonetheless, the experimental results revealed an inverse correlation between the TMA content and mortality. The two groups with the highest TMA concentrations (E_AT and E_TT) had the lowest mortality rates, whereas those with the lowest TMA concentrations (E_AA and C_AA) had relatively high mortality rates. These observations support the inference of an inverse association between the TMA content of egg yolks and chick mortality. However, we did not perform necropsy or microbial testing on dead chicks, so the specific causes of mortality could not be confirmed.

## 5. Conclusions

This study was conducted to determine the effects of increasing the TMA content in hatching eggs on chick quality. The results demonstrated that increasing the TMA content in hatching eggs within a certain range significantly enhanced chick quality at hatching (e.g., based on the Pasgar score and perfect score rate), while concurrently decreasing chick mortality, although this decrease was not significant. Compared with traditional hatchery egg disinfection techniques, this innovative approach utilizes increased concentrations of TMA in the egg yolk to achieve an “inside-out” antibacterial effect. This contributes to enhanced chick quality and has potential in improving hatchability, offering a novel, biologically grounded strategy for optimizing hatching outcomes.

## Figures and Tables

**Table 1:** Composition and nutrient levels of the basal diet (%, air-dry basis).

Items		Items	
Ingredients (%)		Nutrient levels ^3^	
Corn	62.50	Metabolic energy (MJ/Kg)	11.42
Soybean meal	26.20	Total methionine	0.42
Soy oil	1.00	Crude protein	16.04
Limestone	9.58	Total lysine	0.81
Sodium chloride	0.30	Total threonine	0.60
Choline chloride	0.10	Ca	3.64
Vitamin premix ^1^	0.02	Available phosphorus	0.35
Trace mineral premix ^2^	0.30		

^1^ Provided per kg of feed: Vitamin A, 14,000 IU; Vitamin D3, 4500 IU; Vitamin E, 70 mg; Vitamin K3, 3.5 mg; Vitamin B1, 3.0 mg; Vitamin B2, 6.9 mg; Vitamin B6, 4.6 mg; Vitamin B12, 0.025 mg; Panto-thenic acid, 10 mg; Niacin 35 mg; Folic acid, 1 mg; Vegetable acid 100 mg; ^2^ Provided per kg of feed: Fe, 60 mg; Cu, 15 mg; Mn, 120 mg; Zn, 110 mg; I, 1.2 mg. ^3^ Calculated values.

**Table 2:** Pasgar Scoring Criteria for Chick Quality Assessment.

Criterion	Ideal Condition (Score = 10)	Scoring Rule
Vitality	Alert and responsive	1 point deducted for reduced activity or unresponsiveness
Navel closure	Complete closure, well healed	1 point deducted for incomplete closure or inflammation
Leg condition	Normal appearance, no swelling or redness	1 point deducted for swelling, redness, or deformity
Beak development	Proper shape, no deformities	1 point deducted for any visible deformity
Yolk sac absorption	Minimal residual yolk, fully absorbed	1 point deducted for visible or unabsorbed yolk sac

The maximum total score is 10. Chicks scoring below 5 were considered to have unacceptable quality.

**Table 3:** Comparison table of egg hatchability performance and Pasgar score for chicks.

Items		Hatching Rate (%)	Rate of Pasgar Perfect Score (%)	Abnormal Vitality Rate (%)	Abnormal Rate of Legs (%)	Beak AbnorMality Rate (%)	Navel AbnorMality Rate (%)	Belly AbnorMality Rate (%)
Control group	C_AA	80.49	47.06	20.59	0	0	44.12	2.94
	C_AT	80.67	62.27	3.64	0	1.82	21.82	12.73
	C_TT	84.27	78.08	2.74	0	0	17.81	8.22
Experiment group	E_AA	82.50	45.50	9.38	0	0	34.38	6.88
	E_AT	86.02	67.09	6.33	0	0	14.18	6.33
	E_TT	78.50	80.72	2.41	0	0	12.05	6.02

**Table 4:** Comparison of Hatchability and Chick Quality Between High- and Low-TMA Groups.

Group	TMA Content (μg/g)	Hatching Rate (%)	Pasgar Score	Rate of Pasgar Perfect Score (%)	Abnormal Vitality Rate (%)	Navel Abnormality Rate (%)	Belly AbnorMality Rate (%)
Low-TMA	3.28 ± 0.15 ^b^	81.22 ± 0.64	9.26 ± 0.07 ^b^	51.61 ± 5.35 ^b^	11.20 ± 4.98	33.44 ± 6.45	7.52 ± 2.84
High-TMA	6.32 ± 0.55 ^a^	82.93 ± 2.27	9.63 ± 0.04 ^a^	75.30 ± 4.17 ^a^	3.83 ± 1.26	14.68 ± 1.68	6.86 ± 0.69
*p*-value	<0.001	0.535	0.001	0.028	0.273	0.092	0.841

^a,b^ In the same column, means with different superscripts indicate significant differences (*p* < 0.05). Data are expressed as mean ± SE. Groups were classified based on average TMA content in yolk. High-TMA ≥ 4 μg/g; Low-TMA ≤ 4 μg/g.

## Data Availability

The relevant data of this study are included in the article. If you have any further questions, please contact the first author of the article.

## Abbreviations

TMA	Trimethylamine
TMAO	Trimethylamine-N-oxide

## References

[1] Da Silva Oliveira G., McManus C., Salgado C.B., dos Santos V.M. (2022). Effects of Sanitizers on Microbiological Control of Hatching Eggshells and Poultry Health during Embryogenesis and Early Stages after Hatching in the Last Decade. Animals.

[2] Da Silva Oliveira G., McManus C., Vale I.R.R., dos Santos V.M. (2024). Obtaining Microbiologically Safe Hatching Eggs from Hatcheries: Using Essential Oils for Integrated Sanitization Strategies in Hatching Eggs, Poultry Houses and Poultry. Pathogens.

[3] Olsen R., Kudirkiene E., Thøfner I., Pors S., Karlskov-Mortensen P., Li L., Papasolomontos S., Angastiniotou C., Christensen J. (2017). Impact of Egg Disinfection of Hatching Eggs on the Eggshell Microbiome and Bacterial Load. Poult. Sci..

[4] Melo E.F., Clímaco W.L.S., Triginelli M.V., Vaz D.P., De Souza M.R., Baião N.C., Pompeu M.A., Lara L.J.C. (2019). An Evaluation of Alternative Methods for Sanitizing Hatching Eggs. Poult. Sci..

[5] Réhault-Godbert S., Hincke M., Guabiraba R., Guyot N., Gautron J. (2022). Innate Defenses of the Avian Egg. Avian Immunol..

[6] Heier B.T., Jarp J. (2001). An Epidemiological Study of the Hatchability in Broiler Breeder Flocks. Poult. Sci..

[7] Mazanko M.S., Gorlov I.F., Prazdnova E.V., Makarenko M.S., Usatov A.V., Bren A.B., Chistyakov V.A., Tutelyan A.V., Komarova Z.B., Mosolova N.I. (2018). Bacillus Probiotic Supplementations Improve Laying Performance, Egg Quality, Hatching of Laying Hens, and Sperm Quality of Roosters. Probiotics Antimicrob. Proteins.

[8] Wlazlo L., Drabik K., Al-Shammari K.I.A., Batkowska J., Nowakowicz-Debek B., Gryzińska M. (2020). Use of Reactive Oxygen Species (Ozone, Hydrogen Peroxide) for Disinfection of Hatching Eggs. Poult. Sci..

[9] Motola G., Hafez H.M., Brüggemann-Schwarze S. (2023). Assessment of Three Alternative Methods for Bacterial Disinfection of Hatching Eggs in Comparison with Conventional Approach in Commercial Broiler Hatcheries. PLoS ONE.

[10] Cadirci S. (2009). Disinfection of Hatching Eggs by Formaldehyde Fumigation—A Review. Eur. Poult. Sci..

[11] Wang J., Yue H.Y., Xia Z.Q., Wu S.G., Zhang H.J., Ji F., Xu L., Qi G.H. (2012). Effect of Dietary Choline Supplementation under Different Flavin-Containing Monooxygenase 3 Genotypes on Trimethylamine Metabolism in Laying Hens. Poult. Sci..

[12] Goh Y.Q., Cheam G., Wang Y. (2021). Understanding Choline Bioavailability and Utilization: First Step Toward Personalizing Choline Nutrition. J. Agric. Food Chem..

[13] Honkatukia M., Reese K., Preisinger R., Tuiskula-Haavisto M., Weigend S., Roito J., Mäki-Tanila A., Vilkki J. (2005). Fishy Taint in Chicken Eggs Is Associated with a Substitution within a Conserved Motif of the FMO3 Gene. Genomics.

[14] Li X., Huang M., Song J., Shi X., Chen X., Yang F., Pi J., Zhang H., Xu G., Zheng J. (2019). Analysis of Fishy Taint in Duck Eggs Reveals the Causative Constituent of the Fishy Odor and Factors Affecting the Perception Ability of This Odor. Poult. Sci..

[15] Shi X., Li X., Li X., He Z., Chen X., Song J., Zeng L., Liang Q., Li J., Xu G. (2022). Antibacterial Properties of TMA against Escherichia Coli and Effect of Temperature and Storage Duration on TMA Content, Lysozyme Activity and Content in Eggs. Foods.

[16] Li X., Song J., Shi X., Huang M., Liu L., Yi G., Yang N., Xu G., Zheng J. (2022). FMO3 Deficiency of Duck Leads to Decreased Lipid Deposition and Increased Antibacterial Activity. J. Anim. Sci. Biotechnol..

[17] Boerjan M.L. (2002). Programs for Single Stage Incubation and Chick Quality. Avian Poult. Biol. Rev..

[18] Mo F., Zheng J., Wang P., Lian L., Yi G., Xu G., Yang N. (2013). Quail FMO3 Gene Cloning, Tissue Expression Profiling, Polymorphism Detection and Association Analysis with Fishy Taint in Eggs. PLoS ONE.

[19] Li X., Yuan G., Chen X., Guo Y., Yang N., Pi J., Zhang H., Zheng J. (2018). Fishy Odor and TMA Content Levels in Duck Egg Yolks. J. Food Sci..

[20] Fennema D., Phillips I.R., Shephard E.A. (2016). Trimethylamine and Trimethylamine *N*-Oxide, a Flavin-Containing Monooxygenase 3 (FMO3)-Mediated Host-Microbiome Metabolic Axis Implicated in Health and Disease. Drug Metab. Dispos..

[21] Zhang W.-Q., Wang Y.-J., Zhang A., Ding Y.-J., Zhang X.-N., Jia Q.-J., Zhu Y.-P., Li Y.-Y., Lv S.-C., Zhang J.-P. (2021). TMA/TMAO in Hypertension: Novel Horizons and Potential Therapies. J. Cardiovasc. Transl. Res..

[22] Arias N., Arboleya S., Allison J., Kaliszewska A., Higarza S.G., Gueimonde M., Arias J.L. (2020). The Relationship between Choline Bioavailability from Diet, Intestinal Microbiota Composition, and Its Modulation of Human Diseases. Nutrients.

[23] Canyelles M., Tondo M., Cedó L., Farràs M., Escolà-Gil J.C., Blanco-Vaca F. (2018). Trimethylamine N-Oxide: A Link among Diet, Gut Microbiota, Gene Regulation of Liver and Intestine Cholesterol Homeostasis and HDL Function. Int. J. Mol. Sci..

[24] Coutinho-Wolino K.S., de F. Cardozo L.F.M., de Oliveira Leal V., Mafra D., Stockler-Pinto M.B. (2021). Can Diet Modulate Trimethylamine N-Oxide (TMAO) Production? What Do We Know so Far?. Eur. J. Nutr..

[25] Ward A.K., Classen H.L., Buchanan F.C. (2009). Fishy-Egg Tainting Is Recessively Inherited When Brown-Shelled Layers Are Fed Canola Meal. Poult. Sci..

[26] Almas K., Al-Lafi T.R. (1995). The Natural Toothbrush. World Health Forum.

[27] Jones S.E., Ho L., Rees C.A., Hill J.E., Nodwell J.R., Elliot M.A. (2017). Streptomyces Exploration Is Triggered by Fungal Interactions and Volatile Signals. eLife.

[28] Aygun A., Sert D., Copur G. (2012). Effects of Propolis on Eggshell Microbial Activity, Hatchability, and Chick Performance in Japanese Quail (*Coturnix coturnix japonica*) Eggs. Poult. Sci..

[29] Jin J., Zhou Q., Lan F., Li J., Yang N., Sun C. (2022). Microbial Composition of Egg Component and Its Association with Hatchability of Laying Hens. Front. Microbiol..

[30] Tebrün W., Motola G., Hafez M.H., Bachmeier J., Schmidt V., Renfert K., Reichelt C., Brüggemann-Schwarze S., Pees M. (2020). Preliminary Study: Health and Performance Assessment in Broiler Chicks Following Application of Six Different Hatching Egg Disinfection Protocols. PLoS ONE.

[31] Meisinger B.D. (2022). Investigation of the Impacts of Hatchery Practices on Intestinal Microflora of Late-Stage Embryos and Early Post-Hatch Chicks. Ph.D. Thesis.

[32] Chousalkar K.K., Khan S., McWhorter A.R. (2021). Microbial Quality, Safety and Storage of Eggs. Curr. Opin. Food Sci..

[33] Maatjens C.M., van Roovert-Reijrink I.A.M., Engel B., Van der Pol C.W., Kemp B., Van den Brand H. (2016). Temperature during the Last Week of Incubation. I. Effects on Hatching Pattern and Broiler Chicken Embryonic Organ Development. Poult. Sci..

[34] Rezaee M.S., Liebhart D., Hess C., Hess M., Paudel S. (2021). Bacterial Infection in Chicken Embryos and Consequences of Yolk Sac Constitution for Embryo Survival. Vet. Pathol..

[35] King’Ori A.M. (2011). Review of the Factors That Influence Egg Fertility and Hatchability in Poultry. Int. J. Poult. Sci..

[36] Julian R.J. (2005). Production and Growth Related Disorders and Other Metabolic Diseases of Poultry—A Review. Vet. J..

[37] Yerpes M., Llonch P., Manteca X. (2020). Factors Associated with Cumulative First-Week Mortality in Broiler Chicks. Animals.

[38] Gadde U., Rathinam T., Lillehoj H.S. (2015). Passive Immunization with Hyperimmune Egg-Yolk IgY as Prophylaxis and Therapy for Poultry Diseases—A Review. Anim. Health Res. Rev..

[39] Rahman S. (2023). Egg Yolk Antibody-IgY. Handbook of Egg Science and Technology.

[40] Hatta H., Ozeki M., Tsuda K. (2018). Egg Yolk Antibody IgY and Its Application. Hen Eggs.

